# 1-[4-(Diamino­methyl­eneamino­sulfon­yl)phenyl­iminiometh­yl]-2-naphtholate *N*,*N*-dimethyl­formamide disolvate

**DOI:** 10.1107/S1600536808018710

**Published:** 2008-06-28

**Authors:** Hoda El-Ghamry, Raafat Issa, Kamal El-Baradie, Keiko Isagai, Shigeyuki Masaoka, Ken Sakai

**Affiliations:** a Department of Chemistry, Faculty of Science, Tanta University, Tanta, Egypt; b Department of Chemistry, Faculty of Science, Kyushu University, Hakozaki 6-10-1, Higashi-ku, Fukuoka 812-8581, Japan

## Abstract

The asymmetric unit the title compound, C_18_H_16_N_4_O_3_S·2C_3_H_7_NO, contains a mol­ecule in a zwitterionic form with a deprotonated hydroxyl group and an iminium group, and two dimethyl­formamide solvent mol­ecules. The dihedral angles of the guanidine group and the naphthyl ring system with respect to the central benzene ring are 76.04 (7) and 3.45 (9)°, respectively. The conformation of the mol­ecule may be influenced, in part, by two intra­molecular hydrogen bonds, while in the crystal structure, inter­molecular hydrogen bonds form one-dimensional chains along [010].

## Related literature

For related literature, see: Arestrup (1999 ▶); Bergant *et al.* (1993 ▶); Boghaei *et al.* (2000 ▶); Esposito *et al.* (2000 ▶); Ganolkar (1985 ▶); Hao & Shen (2000 ▶); Jain & Chaturvedi (1977 ▶); Jeewoth *et al.* (2000 ▶); Johnson *et al.* (1982 ▶); Kwiatkowski *et al.* (2003 ▶); Lal (1979 ▶); Maki & Hashimato (1952 ▶); Papie *et al.* (1994 ▶); Raman *et al.* (2003 ▶); Srinivasan *et al.* (1986 ▶); Wu & Lu (2003 ▶); Tantaru *et al.* (2002 ▶).
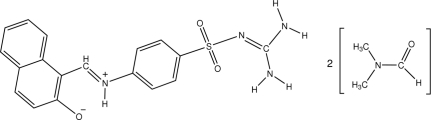


## Experimental

### 

#### Crystal data

C_18_H_16_N_4_O_3_S·2C_3_H_7_NO
*M* *_r_* = 514.60
Monoclinic, 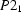

*a* = 8.5910 (11) Å
*b* = 9.9101 (12) Å
*c* = 15.1762 (19) Å
β = 106.327 (1)°
*V* = 1240.0 (3) Å^3^
*Z* = 2
Mo *K*α radiation
μ = 0.18 mm^−1^
*T* = 100 (2) K
0.20 × 0.20 × 0.20 mm

#### Data collection

Bruker SMART APEX CCD-detector diffractometer
Absorption correction: multi-scan (*SADABS*; Sheldrick, 1996 ▶) *T*_min_ = 0.775, *T*_max_ = 0.965
13950 measured reflections
5450 independent reflections
5190 reflections with *I* > 2σ(*I*)
*R*_int_ = 0.026

#### Refinement

*R*[*F* ^2^ > 2σ(*F* ^2^)] = 0.036
*wR*(*F* ^2^) = 0.091
*S* = 1.08
5450 reflections
329 parameters
1 restraint
H-atom parameters constrained
Δρ_max_ = 0.47 e Å^−3^
Δρ_min_ = −0.22 e Å^−3^
Absolute structure: Flack (1983), 2555 Friedel pairs
Flack parameter: −0.03 (6)

### 

Data collection: *APEX2* (Bruker, 2006 ▶); cell refinement: *SAINT* (Bruker, 2004 ▶); data reduction: *SAINT*; program(s) used to solve structure: *SHELXS97* (Sheldrick, 2008 ▶); program(s) used to refine structure: *SHELXL97* (Sheldrick, 2008 ▶); molecular graphics: *KENX* (Sakai, 2004 ▶); software used to prepare material for publication: *SHELXL97*, *TEXSAN* (Molecular Structure Corporation, 2001 ▶), *KENX* and *ORTEPII* (Johnson, 1976 ▶).

## Supplementary Material

Crystal structure: contains datablocks global, I. DOI: 10.1107/S1600536808018710/lh2634sup1.cif

Structure factors: contains datablocks I. DOI: 10.1107/S1600536808018710/lh2634Isup2.hkl

Additional supplementary materials:  crystallographic information; 3D view; checkCIF report

## Figures and Tables

**Table 1:** Hydrogen-bond geometry (Å, °)

*D*—H⋯*A*	*D*—H	H⋯*A*	*D*⋯*A*	*D*—H⋯*A*
N2—H2*B*⋯O2	0.88	2.14	2.781 (2)	129
N4—H4*A*⋯O3	0.88	1.86	2.560 (2)	135
N1—H1*B*⋯O2^i^	0.88	2.14	2.959 (2)	155
N1—H1*A*⋯O5^ii^	0.88	2.07	2.874 (2)	152
N2—H2*A*⋯O5^ii^	0.88	2.16	2.943 (2)	148

Symmetry codes: (i) 
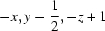
; (ii) 
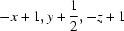
.

**Fig. 1.**
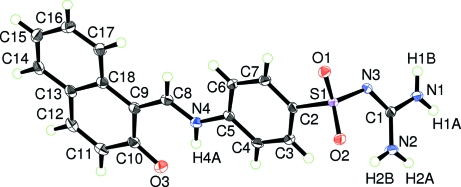
The molecular structure of (I) showing the atom-labeling scheme. Displacement ellipsoids are drawn at the 50% probability level. The solvent molecules are not shown.

**Fig. 2.**
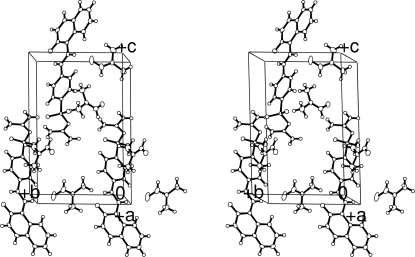
A stereoview for the crystal packing of (I).

## supplementary crystallographic information

### Comment

Schiff bases are considered a very important class of ligands as they easily form stable complexes with most transition metals. Moreover, Schiff bases and their metal complexes are becoming increasingly important as biochemical (Johnson *et al.*, 1982), analytical (Hao & Shen, 2000; Tantaru *et al.*, 2002), industrial (Srinivasan *et al.*, 1986) reagents and redox catalysts (Jeewoth *et al.*, 2000; Boghaei *et al.*, 2000; Wu & Lu, 2003; Kwiatkowski *et al.*, 2003) as well as pigment dyes (Maki & Hashimato, 1952; Papie *et al.*, 1994). What appears more important is that Schiff bases and their metal complexes are useful in biological and pharmaceutical applications (Ganolkar, 1985; Bergant *et al.*, 1993; Raman *et al.*, 2003). Sulfonamides are also the oldest class of antimicrobials and are still the drug of choice forroup and protonated amino group many diseases such as cancer and tuberculosis (Arestrup, 1999; Esposito *et al.*, 2000). A number of references are now available to show that the condensation products of sulfonamides with aldehydes and ketones are also biologically active and have a good ability for complexation (Jain & Chaturvedi, 1977; Lal, 1979). In this paper, we report the synthesis and crystal structure of the title compound (I).

The asymmetric unit of (I) consists of one independent molecule of a zwitterion and two molecules of dimethylformamide. The oxygen atom attached to the naphthyl group (O3) is found to be a deprotonated form of hydroxyl group. The imine unit connecting the naphthyl and phenyl groups is in a protonated form, giving an iminium unit. Two intramolecular hydrogen bonds (N4—H4A···O3 and N2—H2B···O2; Table 2) are formed to stabilize the conformation of the molecule. Three intermolecular hydrogen bonds also take part in stabilizing the conformation together with the crystal packing of the compound [N1—H1B···O2(i), N1—H1A···O5(ii), N2—H2A···O5(ii); symmetry operation (i): –*x*, y-0.5, 1-*z*, (ii): –*x*+1, 0.5+y, 1-*z*]. The guanidine unit, consisting of C1 and N1—N3 atoms, forms a planar geometry and is canted with respect to the central phenyl ring at an angle of 76.04 (7) °. The C=N double bond character of the guanidine moiety is fully delocalized over the unit as shown by the similar C—N distances within the unit. On the other hand, the naphthyl plane is declined only by 3.45 (9) ° with regard to the phenyl ring. The iminium unit, consisting of atoms C8 and N4 [C8—N4 = 1.322 (2) Å], is nearly coplanar to the naphthyl plane, as can be seen by the torsion angles about the C8—C9 axis, *i.e.*, N4—C8—C9—C10 = -1.0 (3) and N4—C8—C9—C18 = 178.27 (17) °. Good planarity is exhibited by the guanidine and phenyl moieties, while the naphthyl moiety shows a deviation from the planar geometry, where the ten atom r.m.s deviation estimated in the best plane calculation is 0.024 Å. No obvious stacking interaction is found in the crystal (see Fig. 2).

### Experimental

Compound (I) was prepared as follows. A hot methanolic solution of 2-hydroxy-1-naphthaldehyde (0.9 mmol, 0.154 g) was added to a methanolic solution of sulfaguanidine (0.9 mmol, 0.192 g). The resulting solution was then refluxed with stirring for 2 h during which a yellow precipitate deposited. The precipitate was then filtered off from hot solution and then air-dried (yield: 85%). Analysis calculated for C_18_H_16_N_4_O_3_S: C, 58.69; H, 4.34; N, 15.21; S, 8.69% found: C, 59.16; H, 4.39; N, 14.94; S, 8.76%. IR (ν, cm^-1^): 3393 (*m*), 3316 (*m*), 3167 (w), 1614 (*s*), 1591 (*s*), 1580 (sh), 1540 (*s*),1514 (*s*), 1406 (*m*), 1347 (*m*), 1316 (*m*), 1302 (w), 1295 (w), 1255 (*s*), 1175 (*m*), 1128 (*s*), 1092 (*s*), 1060 (*s*), 1042 (w), 1014 (*m*), 989 (w), 978 (w), 966 (*m*), 964 (w), 835 (*s*), 822 (*s*), 811 (w), 743 (*s*), 727 (w), 686 (*m*), 608 (*s*), 558 (*s*), 539 (*s*), 475 (*s*), 432 (*s*), 409 (*s*). A good quality single-crystal of (I) was prepared by vapour diffusion method as follow. Compound (I) was dissolved in a minimum amount of *N*,*N*-dimethylformamide and the solution was left in refrigerator in the presence of ether pool. Upon leaving the solution for 3 days, it gradually raised its volume to give crystals suitable for X-ray diffraction analysis.

### Refinement

All H atoms were placed in idealized positions (methyl C—H = 0.98 Å, aromatic C—H = 0.95 Å, and N—H = 0.88 Å), and included in the refinement in a riding-model approximation, with *U*_iso_(H) = 1.5U_eq_(methyl C) and *U*_iso_(H) = 1.2U_eq_(aromatic C and N). In the final difference Fourier map, the highest peak was located 0.86 Å from atom C2. The deepest hole was located 0.51 Å from atom S1.

### Crystal data

**Table:**

C_18_H_16_N_4_O_3_S·2C_3_H_7_NO	*F*_000_ = 544
*M**_r_* = 514.60	*D*_x_ = 1.378 Mg m^−^^3^
Monoclinic, *P*2_1_	Mo *K*α radiation λ = 0.71073 Å
Hall symbol: P 2yb	Cell parameters from 7706 reflections
*a* = 8.5910 (11) Å	θ = 2.5–28.3º
*b* = 9.9101 (12) Å	µ = 0.18 mm^−^^1^
*c* = 15.1762 (19) Å	*T* = 100 (2) K
β = 106.327 (1)º	Cube, yellow
*V* = 1240.0 (3) Å^3^	0.20 × 0.20 × 0.20 mm
*Z* = 2	

### Data collection

**Table:**

Bruker SMART APEX CCD-detector diffractometer	5450 independent reflections
Radiation source: sealed tube	5190 reflections with *I* > 2σ(*I*)
Monochromator: graphite	*R*_int_ = 0.026
*T* = 100(2) K	θ_max_ = 27.1º
φ and ω scans	θ_min_ = 2.5º
Absorption correction: multi-scan(SADABS; Sheldrick, 1996)	*h* = −10→11
*T*_min_ = 0.775, *T*_max_ = 0.965	*k* = −12→12
13950 measured reflections	*l* = −19→19

### Refinement

**Table:**

Refinement on *F*^2^	Hydrogen site location: inferred from neighbouring sites
Least-squares matrix: full	H-atom parameters constrained
*R*[*F*^2^ > 2σ(*F*^2^)] = 0.036	*w* = 1/[σ^2^(*F*_o_^2^) + (0.0491*P*)^2^ + 0.3529*P*] where *P* = (*F*_o_^2^ + 2*F*_c_^2^)/3
*wR*(*F*^2^) = 0.091	(Δ/σ)_max_ = 0.001
*S* = 1.08	Δρ_max_ = 0.47 e Å^−^^3^
5450 reflections	Δρ_min_ = −0.22 e Å^−^^3^
329 parameters	Extinction correction: none
1 restraint	Absolute structure: Flack (1983), 2555 Friedel pairs
Primary atom site location: structure-invariant direct methods	Flack parameter: −0.03 (6)
Secondary atom site location: difference Fourier map	

### Special details

**Table:**

Experimental. The first 50 frames were rescanned at the end of data collection to evaluate any possible decay phenomenon. Since it was judged to be negligible, no decay correction was applied to the data.
Geometry. All e.s.d.'s (except the e.s.d. in the dihedral angle between two l.s. planes) are estimated using the full covariance matrix. The cell e.s.d.'s are taken into account individually in the estimation of e.s.d.'s in distances, angles and torsion angles; correlations between e.s.d.'s in cell parameters are only used when they are defined by crystal symmetry. An approximate (isotropic) treatment of cell e.s.d.'s is used for estimating e.s.d.'s involving l.s. planes.Least-squares planes (*x*,*y*,*z* in crystal coordinates) and deviations from them (* indicates atom used to define plane)5.2137 (0.0068) *x* - 0.3949 (0.0103) *y* + 8.9719 (0.0123) *z* = 4.0913 (0.0088)* -0.0014 (0.0004) N1 * -0.0016 (0.0005) N2 * -0.0016 (0.0005) N3 * 0.0047 (0.0014) C1Rms deviation of fitted atoms = 0.0027- 6.1727 (0.0045) *x* + 6.3206 (0.0058) *y* + 7.1057 (0.0101) *z* = 8.7939 (0.0072)Angle to previous plane (with approximate e.s.d.) = 76.04 (0.07)* 0.0058 (0.0013) C2 * 0.0060 (0.0013) C3 * -0.0134 (0.0013) C4 * 0.0091 (0.0013) C5 * 0.0025 (0.0013) C6 * -0.0100 (0.0013) C7 0.0707 (0.0027) N4 0.1033 (0.0034) C8Rms deviation of fitted atoms = 0.0085- 6.1442 (0.0026) *x* + 6.5659 (0.0029) *y* + 6.2926 (0.0042) *z* = 8.2691 (0.0057)Angle to previous plane (with approximate e.s.d.) = 3.45 (0.09)* 0.0103 (0.0013) N4 * -0.0338 (0.0015) C8 * -0.0052 (0.0016) C9 * 0.0523 (0.0016) C10 * 0.0115 (0.0016) C11 * -0.0294 (0.0016) C12 * -0.0226 (0.0017) C13 * -0.0027 (0.0016) C14 * 0.0199 (0.0017) C15 * 0.0248 (0.0017) C16 * -0.0069 (0.0018) C17 * -0.0184 (0.0017) C18Rms deviation of fitted atoms = 0.0240- 6.1272 (0.0029) *x* + 6.5698 (0.0029) *y* + 6.3590 (0.0070) *z* = 8.3630 (0.0094)Angle to previous plane (with approximate e.s.d.) = 0.32 (0.06)* -0.0161 (0.0015) C9 * 0.0447 (0.0015) C10 * 0.0118 (0.0016) C11 * -0.0248 (0.0016) C12 * -0.0211 (0.0017) C13 * 0.0034 (0.0016) C14 * 0.0230 (0.0017) C15 * 0.0202 (0.0017) C16 * -0.0161 (0.0016) C17 * -0.0248 (0.0016) C18 - 0.0128 (0.0027) N4 - 0.0526 (0.0025) C8 0.1229 (0.0022) O3Rms deviation of fitted atoms = 0.0230
Refinement. Refinement of *F*^2^ against ALL reflections. The weighted *R*-factor *wR* and goodness of fit *S* are based on *F*^2^, conventional *R*-factors *R* are based on *F*, with *F* set to zero for negative *F*^2^. The threshold expression of *F*^2^ > σ(*F*^2^) is used only for calculating *R*-factors(gt) *etc*. and is not relevant to the choice of reflections for refinement. *R*-factors based on *F*^2^ are statistically about twice as large as those based on *F*, and *R*- factors based on ALL data will be even larger.

### Fractional atomic coordinates and isotropic or equivalent isotropic displacement parameters (Å^2^)

**Table:**

	*x*	*y*	*z*	*U*_iso_*/*U*_eq_	
S1	−0.11893 (5)	0.64754 (4)	0.56177 (3)	0.01452 (10)	
O1	−0.24764 (16)	0.55840 (14)	0.56814 (9)	0.0193 (3)	
O2	−0.16593 (16)	0.78608 (13)	0.53549 (9)	0.0194 (3)	
O3	0.57291 (18)	0.81413 (15)	1.04536 (10)	0.0256 (3)	
O4	−0.0111 (3)	0.32634 (18)	0.94460 (18)	0.0650 (7)	
O5	0.72580 (19)	0.28022 (15)	0.68340 (11)	0.0285 (3)	
N1	0.1366 (2)	0.56994 (16)	0.40154 (11)	0.0206 (3)	
N2	0.0756 (2)	0.77841 (17)	0.44614 (12)	0.0218 (4)	
N3	−0.02653 (19)	0.57624 (15)	0.49661 (10)	0.0165 (3)	
N4	0.3407 (2)	0.68157 (15)	0.93727 (11)	0.0182 (3)	
N5	0.0840 (2)	0.11583 (17)	0.93544 (12)	0.0260 (4)	
N6	0.5259 (2)	0.43169 (17)	0.68153 (12)	0.0219 (4)	
C1	0.0599 (2)	0.6446 (2)	0.45009 (11)	0.0163 (3)	
C2	0.0195 (2)	0.6549 (2)	0.67283 (11)	0.0152 (3)	
C3	0.1424 (2)	0.75172 (18)	0.69345 (13)	0.0186 (4)	
C4	0.2502 (2)	0.75575 (19)	0.78083 (13)	0.0190 (4)	
C5	0.2327 (2)	0.66596 (18)	0.84865 (12)	0.0169 (4)	
C6	0.1098 (2)	0.56887 (18)	0.82732 (13)	0.0183 (4)	
C7	0.0047 (2)	0.56322 (19)	0.73931 (12)	0.0180 (4)	
C8	0.3396 (2)	0.60541 (19)	1.00860 (13)	0.0184 (4)	
C9	0.4473 (2)	0.62620 (18)	1.09660 (13)	0.0176 (4)	
C10	0.5652 (2)	0.73244 (19)	1.11006 (13)	0.0197 (4)	
C11	0.6790 (2)	0.74704 (19)	1.19945 (14)	0.0222 (4)	
C12	0.6721 (2)	0.6666 (2)	1.27010 (13)	0.0224 (4)	
C13	0.5510 (2)	0.56323 (19)	1.26085 (13)	0.0191 (4)	
C14	0.5425 (2)	0.4853 (2)	1.33700 (13)	0.0223 (4)	
C15	0.4250 (3)	0.3869 (2)	1.32851 (15)	0.0248 (4)	
C16	0.3137 (2)	0.3655 (2)	1.24287 (15)	0.0236 (4)	
C17	0.3208 (2)	0.4402 (2)	1.16690 (13)	0.0221 (4)	
C18	0.4381 (2)	0.54178 (19)	1.17358 (13)	0.0179 (4)	
C19	−0.0038 (3)	0.2224 (2)	0.90039 (19)	0.0373 (6)	
C20	0.1016 (3)	0.0011 (2)	0.87992 (17)	0.0332 (5)	
C21	0.1793 (4)	0.1120 (3)	1.03111 (17)	0.0470 (7)	
C22	0.5933 (3)	0.3374 (2)	0.64427 (16)	0.0282 (5)	
C23	0.5988 (3)	0.4800 (2)	0.77380 (15)	0.0324 (5)	
C24	0.3774 (3)	0.4992 (3)	0.62885 (18)	0.0399 (6)	
H1A	0.1939	0.6095	0.3691	0.025*	
H1B	0.1297	0.4814	0.4021	0.025*	
H2A	0.1342	0.8135	0.4128	0.026*	
H2B	0.0274	0.8314	0.4769	0.026*	
H4A	0.4136	0.7462	0.9456	0.022*	
H3	0.1522	0.8145	0.6480	0.022*	
H4	0.3362	0.8196	0.7946	0.023*	
H6	0.0983	0.5070	0.8729	0.022*	
H7	−0.0777	0.4963	0.7244	0.022*	
H8	0.2632	0.5339	1.0002	0.022*	
H11	0.7606	0.8145	1.2090	0.027*	
H12	0.7499	0.6787	1.3280	0.027*	
H14	0.6186	0.5005	1.3950	0.027*	
H15	0.4201	0.3347	1.3802	0.030*	
H16	0.2318	0.2988	1.2366	0.028*	
H17	0.2449	0.4224	1.1091	0.026*	
H19	−0.0651	0.2194	0.8377	0.045*	
H20A	0.0285	0.0114	0.8178	0.050*	
H20B	0.0744	−0.0819	0.9075	0.050*	
H20C	0.2139	−0.0040	0.8768	0.050*	
H21A	0.1438	0.1844	1.0649	0.071*	
H21B	0.2943	0.1243	1.0351	0.071*	
H21C	0.1642	0.0246	1.0578	0.071*	
H22	0.5385	0.3102	0.5834	0.034*	
H23A	0.6502	0.5676	0.7713	0.049*	
H23B	0.6807	0.4153	0.8069	0.049*	
H23C	0.5148	0.4898	0.8057	0.049*	
H24A	0.3318	0.4510	0.5710	0.060*	
H24B	0.4024	0.5923	0.6158	0.060*	
H24C	0.2983	0.4996	0.6645	0.060*	

### Atomic displacement parameters (Å^2^)

**Table:**

	*U*^11^	*U*^22^	*U*^33^	*U*^12^	*U*^13^	*U*^23^
S1	0.0178 (2)	0.01154 (19)	0.01503 (19)	0.00018 (18)	0.00590 (15)	0.00046 (17)
O1	0.0198 (7)	0.0192 (6)	0.0197 (6)	−0.0020 (5)	0.0066 (5)	0.0012 (5)
O2	0.0234 (7)	0.0159 (6)	0.0193 (6)	0.0038 (5)	0.0067 (6)	0.0021 (5)
O3	0.0315 (8)	0.0220 (7)	0.0236 (7)	−0.0062 (6)	0.0079 (6)	0.0012 (6)
O4	0.1020 (18)	0.0211 (9)	0.1044 (19)	0.0118 (10)	0.0820 (16)	0.0082 (10)
O5	0.0310 (8)	0.0228 (7)	0.0381 (9)	0.0026 (6)	0.0202 (7)	0.0000 (7)
N1	0.0297 (9)	0.0127 (7)	0.0241 (8)	0.0003 (7)	0.0154 (7)	0.0010 (6)
N2	0.0305 (9)	0.0127 (8)	0.0286 (9)	−0.0008 (7)	0.0189 (8)	0.0002 (7)
N3	0.0235 (8)	0.0120 (7)	0.0153 (7)	0.0010 (6)	0.0076 (6)	−0.0007 (6)
N4	0.0199 (8)	0.0150 (8)	0.0192 (8)	−0.0021 (6)	0.0045 (6)	−0.0006 (6)
N5	0.0352 (10)	0.0159 (9)	0.0279 (9)	−0.0014 (7)	0.0104 (8)	−0.0012 (6)
N6	0.0219 (8)	0.0188 (8)	0.0255 (9)	−0.0017 (7)	0.0076 (7)	−0.0011 (7)
C1	0.0196 (8)	0.0152 (8)	0.0130 (7)	0.0028 (8)	0.0030 (6)	0.0020 (8)
C2	0.0180 (8)	0.0146 (8)	0.0130 (7)	0.0022 (8)	0.0044 (6)	−0.0016 (8)
C3	0.0251 (10)	0.0147 (9)	0.0177 (9)	−0.0017 (7)	0.0087 (8)	0.0008 (7)
C4	0.0226 (10)	0.0135 (9)	0.0227 (10)	−0.0046 (7)	0.0095 (8)	−0.0034 (7)
C5	0.0209 (8)	0.0140 (9)	0.0170 (8)	0.0020 (7)	0.0071 (7)	−0.0019 (7)
C6	0.0240 (10)	0.0139 (9)	0.0180 (9)	−0.0004 (7)	0.0077 (7)	0.0025 (7)
C7	0.0194 (9)	0.0153 (8)	0.0206 (9)	−0.0014 (7)	0.0078 (7)	0.0003 (7)
C8	0.0191 (9)	0.0155 (8)	0.0211 (9)	0.0001 (7)	0.0064 (7)	−0.0013 (7)
C9	0.0195 (9)	0.0153 (9)	0.0187 (8)	0.0025 (7)	0.0063 (7)	−0.0008 (7)
C10	0.0230 (10)	0.0161 (9)	0.0212 (9)	0.0008 (7)	0.0083 (8)	−0.0031 (7)
C11	0.0202 (10)	0.0189 (10)	0.0270 (10)	−0.0025 (8)	0.0060 (8)	−0.0069 (8)
C12	0.0206 (9)	0.0251 (10)	0.0193 (9)	0.0025 (8)	0.0022 (7)	−0.0069 (8)
C13	0.0210 (9)	0.0173 (9)	0.0197 (9)	0.0060 (7)	0.0070 (7)	−0.0035 (8)
C14	0.0258 (10)	0.0237 (10)	0.0166 (9)	0.0067 (8)	0.0046 (8)	−0.0033 (8)
C15	0.0316 (12)	0.0230 (10)	0.0224 (10)	0.0065 (8)	0.0119 (9)	0.0048 (8)
C16	0.0238 (10)	0.0198 (9)	0.0292 (11)	−0.0006 (8)	0.0105 (9)	0.0015 (8)
C17	0.0228 (10)	0.0209 (10)	0.0206 (9)	−0.0007 (8)	0.0032 (8)	0.0002 (8)
C18	0.0193 (9)	0.0163 (9)	0.0190 (9)	0.0046 (7)	0.0067 (7)	−0.0014 (7)
C19	0.0469 (15)	0.0264 (11)	0.0488 (15)	0.0044 (11)	0.0305 (13)	0.0093 (11)
C20	0.0371 (12)	0.0213 (11)	0.0470 (14)	−0.0036 (9)	0.0210 (11)	−0.0056 (10)
C21	0.0679 (19)	0.0395 (15)	0.0297 (12)	−0.0226 (13)	0.0070 (12)	0.0037 (10)
C22	0.0337 (12)	0.0260 (11)	0.0275 (11)	−0.0041 (9)	0.0127 (9)	0.0000 (9)
C23	0.0342 (12)	0.0307 (12)	0.0328 (12)	−0.0030 (10)	0.0105 (10)	−0.0056 (10)
C24	0.0348 (13)	0.0313 (13)	0.0472 (15)	0.0062 (10)	0.0011 (11)	−0.0024 (11)

### Geometric parameters (Å, °)

**Table:**

S1—O1	1.4397 (14)	N6—C22	1.308 (3)
S1—O2	1.4549 (14)	N6—C23	1.445 (3)
S1—N3	1.5963 (16)	N6—C24	1.464 (3)
S1—C2	1.7702 (17)	N1—H1A	0.8800
O3—C10	1.289 (2)	N1—H1B	0.8800
O4—C19	1.240 (3)	N2—H2A	0.8800
O5—C22	1.261 (3)	N2—H2B	0.8800
N1—C1	1.341 (2)	N4—H4A	0.8800
N2—C1	1.336 (2)	C3—H3	0.9500
N3—C1	1.343 (2)	C4—H4	0.9500
N4—C8	1.322 (2)	C6—H6	0.9500
N4—C5	1.411 (2)	C7—H7	0.9500
C2—C7	1.389 (3)	C8—H8	0.9500
C2—C3	1.396 (3)	C11—H11	0.9500
C3—C4	1.388 (3)	C12—H12	0.9500
C4—C5	1.400 (3)	C14—H14	0.9500
C5—C6	1.398 (3)	C15—H15	0.9500
C6—C7	1.387 (3)	C16—H16	0.9500
C8—C9	1.410 (3)	C17—H17	0.9500
C9—C10	1.435 (3)	C19—H19	0.9500
C9—C18	1.457 (3)	C20—H20A	0.9800
C10—C11	1.439 (3)	C20—H20B	0.9800
C11—C12	1.351 (3)	C20—H20C	0.9800
C12—C13	1.438 (3)	C21—H21A	0.9800
C13—C14	1.409 (3)	C21—H21B	0.9800
C13—C18	1.421 (3)	C21—H21C	0.9800
C14—C15	1.383 (3)	C22—H22	0.9500
C15—C16	1.396 (3)	C23—H23A	0.9800
C16—C17	1.385 (3)	C23—H23B	0.9800
C17—C18	1.408 (3)	C23—H23C	0.9800
N5—C19	1.320 (3)	C24—H24A	0.9800
N5—C20	1.448 (3)	C24—H24B	0.9800
N5—C21	1.453 (3)	C24—H24C	0.9800
			
O1—S1—O2	115.99 (8)	C12—C11—H11	119.4
O1—S1—N3	107.16 (8)	C10—C11—H11	119.4
O2—S1—N3	113.33 (8)	C11—C12—C13	122.34 (18)
O1—S1—C2	106.52 (8)	C11—C12—H12	118.8
O2—S1—C2	106.42 (9)	C13—C12—H12	118.8
N3—S1—C2	106.86 (8)	C14—C13—C18	120.20 (18)
C1—N1—H1A	120.0	C14—C13—C12	120.89 (18)
C1—N1—H1B	120.0	C18—C13—C12	118.90 (17)
H1A—N1—H1B	120.0	C15—C14—C13	120.99 (19)
C1—N2—H2A	120.0	C15—C14—H14	119.5
C1—N2—H2B	120.0	C13—C14—H14	119.5
H2A—N2—H2B	120.0	C14—C15—C16	119.00 (19)
C1—N3—S1	123.17 (13)	C14—C15—H15	120.5
C8—N4—C5	124.29 (16)	C16—C15—H15	120.5
C8—N4—H4A	117.9	C17—C16—C15	120.97 (19)
C5—N4—H4A	117.9	C17—C16—H16	119.5
C19—N5—C20	122.4 (2)	C15—C16—H16	119.5
C19—N5—C21	121.4 (2)	C16—C17—C18	121.32 (18)
C20—N5—C21	116.0 (2)	C16—C17—H17	119.3
C22—N6—C23	122.12 (19)	C18—C17—H17	119.3
C22—N6—C24	120.95 (19)	C17—C18—C13	117.50 (17)
C23—N6—C24	116.83 (19)	C17—C18—C9	123.51 (17)
N2—C1—N1	116.84 (17)	C13—C18—C9	118.98 (17)
N2—C1—N3	127.02 (17)	O4—C19—N5	123.8 (3)
N1—C1—N3	116.14 (17)	O4—C19—H19	118.1
C7—C2—C3	120.29 (16)	N5—C19—H19	118.1
C7—C2—S1	119.38 (14)	N5—C20—H20A	109.5
C3—C2—S1	120.34 (14)	N5—C20—H20B	109.5
C4—C3—C2	119.58 (17)	H20A—C20—H20B	109.5
C4—C3—H3	120.2	N5—C20—H20C	109.5
C2—C3—H3	120.2	H20A—C20—H20C	109.5
C3—C4—C5	120.20 (17)	H20B—C20—H20C	109.5
C3—C4—H4	119.9	N5—C21—H21A	109.5
C5—C4—H4	119.9	N5—C21—H21B	109.5
C6—C5—C4	119.83 (17)	H21A—C21—H21B	109.5
C6—C5—N4	123.21 (16)	N5—C21—H21C	109.5
C4—C5—N4	116.94 (17)	H21A—C21—H21C	109.5
C7—C6—C5	119.71 (16)	H21B—C21—H21C	109.5
C7—C6—H6	120.1	O5—C22—N6	124.6 (2)
C5—C6—H6	120.1	O5—C22—H22	117.7
C6—C7—C2	120.35 (17)	N6—C22—H22	117.7
C6—C7—H7	119.8	N6—C23—H23A	109.5
C2—C7—H7	119.8	N6—C23—H23B	109.5
N4—C8—C9	122.57 (17)	H23A—C23—H23B	109.5
N4—C8—H8	118.7	N6—C23—H23C	109.5
C9—C8—H8	118.7	H23A—C23—H23C	109.5
C8—C9—C10	119.41 (17)	H23B—C23—H23C	109.5
C8—C9—C18	120.43 (17)	N6—C24—H24A	109.5
C10—C9—C18	120.16 (17)	N6—C24—H24B	109.5
O3—C10—C9	122.53 (18)	H24A—C24—H24B	109.5
O3—C10—C11	119.23 (18)	N6—C24—H24C	109.5
C9—C10—C11	118.24 (17)	H24A—C24—H24C	109.5
C12—C11—C10	121.26 (18)	H24B—C24—H24C	109.5
			
O1—S1—N3—C1	156.25 (14)	C18—C9—C10—O3	−176.40 (17)
O2—S1—N3—C1	27.00 (17)	C8—C9—C10—C11	−176.88 (17)
C2—S1—N3—C1	−89.89 (16)	C18—C9—C10—C11	3.8 (3)
S1—N3—C1—N2	−3.9 (3)	O3—C10—C11—C12	177.77 (18)
S1—N3—C1—N1	177.04 (13)	C9—C10—C11—C12	−2.4 (3)
O1—S1—C2—C7	11.77 (17)	C10—C11—C12—C13	−0.6 (3)
O2—S1—C2—C7	136.09 (15)	C11—C12—C13—C14	−176.99 (18)
N3—S1—C2—C7	−102.53 (15)	C11—C12—C13—C18	2.2 (3)
O1—S1—C2—C3	−168.26 (14)	C18—C13—C14—C15	0.1 (3)
O2—S1—C2—C3	−43.94 (16)	C12—C13—C14—C15	179.23 (18)
N3—S1—C2—C3	77.44 (16)	C13—C14—C15—C16	−0.1 (3)
C7—C2—C3—C4	0.2 (3)	C14—C15—C16—C17	0.7 (3)
S1—C2—C3—C4	−179.79 (14)	C15—C16—C17—C18	−1.3 (3)
C2—C3—C4—C5	−2.0 (3)	C16—C17—C18—C13	1.2 (3)
C3—C4—C5—C6	2.3 (3)	C16—C17—C18—C9	−177.82 (18)
C3—C4—C5—N4	−176.20 (17)	C14—C13—C18—C17	−0.6 (3)
C8—N4—C5—C6	1.3 (3)	C12—C13—C18—C17	−179.77 (17)
C8—N4—C5—C4	179.76 (18)	C14—C13—C18—C9	178.46 (17)
C4—C5—C6—C7	−0.8 (3)	C12—C13—C18—C9	−0.7 (3)
N4—C5—C6—C7	177.63 (17)	C8—C9—C18—C17	−2.6 (3)
C5—C6—C7—C2	−1.0 (3)	C10—C9—C18—C17	176.74 (17)
C3—C2—C7—C6	1.4 (3)	C8—C9—C18—C13	178.44 (16)
S1—C2—C7—C6	−178.68 (14)	C10—C9—C18—C13	−2.3 (3)
C5—N4—C8—C9	−178.53 (17)	C20—N5—C19—O4	−174.2 (2)
N4—C8—C9—C10	−1.0 (3)	C21—N5—C19—O4	0.8 (4)
N4—C8—C9—C18	178.27 (17)	C23—N6—C22—O5	−0.3 (3)
C8—C9—C10—O3	2.9 (3)	C24—N6—C22—O5	175.8 (2)

### Hydrogen-bond geometry (Å, °)

**Table:**

*D*—H···*A*	*D*—H	H···*A*	*D*···*A*	*D*—H···*A*
N2—H2B···O2	0.88	2.14	2.781 (2)	129
N4—H4A···O3	0.88	1.86	2.560 (2)	135
N1—H1B···O2^i^	0.88	2.14	2.959 (2)	155
N1—H1A···O5^ii^	0.88	2.07	2.874 (2)	152
N2—H2A···O5^ii^	0.88	2.16	2.943 (2)	148

Symmetry codes: (i) −*x*, *y*−1/2, −*z*+1; (ii) −*x*+1, *y*+1/2, −*z*+1.
